# Effective use of PI3K inhibitor BKM120 and PARP inhibitor Olaparib to treat PIK3CA mutant ovarian cancer

**DOI:** 10.18632/oncotarget.7549

**Published:** 2016-02-21

**Authors:** Dong Wang, Min Wang, Nan Jiang, Yuan Zhang, Xing Bian, Xiaoqing Wang, Thomas M. Roberts, Jean J. Zhao, Pixu Liu, Hailing Cheng

**Affiliations:** ^1^ Cancer Institute, The Second Hospital of Dalian Medical University, Institute of Cancer Stem Cell, Dalian Medical University, Dalian 116044, China; ^2^ Department of Histology and Embryology, Binzhou Medical College, Yantai 264000, China; ^3^ Department of Cancer Biology, Dana-Farber Cancer Institute, Boston, MA 02115, USA; ^4^ Department of Biological Chemistry and Molecular Pharmacology, Harvard Medical School, Boston, MA 02115, USA

**Keywords:** ovarian cancer, BKM120, Olaparib, BRCA, combination therapy

## Abstract

Recent preclinical studies revealed the efficacy of combined use of PI3K inhibitor BKM120 and PARP inhibitor Olaparib in breast and prostate cancers. The current study investigated the effect of such drug combination on ovarian cancer. Here we showed that combined inhibition of PI3K and PARP effectively synergized to inhibit proliferation, survival and invasion in the majority of ovarian cancer cell lines harboring *PIK3CA* mutations, including SKOV3, HEYA8, and IGROV1. Mechanistically, combined treatment of PARP and PI3K inhibitors resulted in an exacerbated DNA damage response and more substantially reduced AKT/mTOR signaling when compared to single-agent. Notably, ovarian cancer cells responsive to the PI3K/PARP combination displayed decreased BRCA1/2 expression upon drug treatment. Furthermore, the effect of the drug combination was corroborated in an intraperitoneal dissemination xenograft mouse model in which SKOV3 ovarian cancer cells responded with significantly decreased BRCA1 expression, suppressed PI3K/AKT signaling and reduced tumor burden. Collectively, our data suggested that combined inhibition of PI3K and PARP may be an effective therapeutic strategy for ovarian cancers with *PIK3CA* mutations and that the accompanied BRCA downregulation following PI3K inhibition could serve as a biomarker for the effective response to PARP inhibition.

## INTRODUCTION

The PI3K pathway is an important signaling network that regulates critical cellular functions including cell growth, proliferation and survival [1, 2]. Somatic mutations in the PIK3CA gene are frequently found in a variety of human cancers. *PIK3CA* mutations mainly occur in the kinase domain (H1047R) and the helical domain (E542K or E545K) of p110α, with H1047R being the most common mutation [1]. These tumor-associated *PIK3CA* mutations result in constitutive activation of p110α and its downstream effector AKT signaling with consequent oncogenic transformation [2]. Recent comprehensive genomic characterization of ovarian cancers revealed that aberrant PI3K pathway activation frequently occurs in a significant fraction of this cancer type [3, 4], justifying further investigation of the PI3K signaling pathway as a major therapeutic target for this challenging disease [5]. A number of PI3K inhibitors have shown significant anti-tumor activities either as single-agents or when used in combination with cytotoxic anti-cancer agents in *in vitro* and *in vivo* models of ovarian cancers [5, 6]. BKM120, a pan-class I PI3K inhibitor currently in Phase I/II clinical trials [8, 9], has demonstrated anti-proliferative, pro-apoptotic, and antitumor activity in a variety of cell lines and xenograft models from cancers with and without aberrant PI3K pathway activation [10, 11]. In addition, PI3K suppression has been shown to impair homologous recombination (HR) in the cellular DNA damage response pathway [12, 13].

The poly (ADP-ribose) polymerase (PARP) inhibitor Olaparib has been recently approved by FDA as the first monotherapy to treat BRCA-mutated advanced ovarian cancer [14]. PARP is involved in surveillance and maintenance of genome integrity and functions as a key molecule in the repair of DNA single-stranded breaks (SSBs) [15]. BRCA proteins are critical for homologous recombination (HR) repair of double-stranded DNA breaks (DSBs) [16]. The function of BRCA1 in HR-mediated repair contributes to its tumor suppressor activity [16]. BRCA-deficient cells appear to be highly sensitive to PARP inhibition, resulting in increased genomic instability and apoptosis [16–18]. The combination of a PI3K inhibitor BKM120 with PARP inhibitor Olaparib has reported to exhibit synergistic therapeutic effects for the treatment of a genetic mouse model of BRCA1-related breast cancers as well as for the treatment of BRCA1-proficient triple negative breast cancers [17]. More recently, combined inhibition of PARP and PI3K was reported to confer increased efficacy in hormone-insensitive advanced prostate cancer with PTEN and p53 co-deficiency [19]. Results from these studies have prompted an urgent need for the clinical investigation of the combined use of PI3K inhibitor and PARP inhibitor. Indeed, Phase I clinical trials of such drug combination are currently enrolling patients with triple-negative breast cancer and high-grade serous ovarian cancers [20]. In the current study, we set out to investigate the inhibitory effect of combination treatment on *PIK3CA* mutated ovarian cancer cells and the underlying mechanisms that account for the therapeutic effect in *in vitro* and *in vivo*. To further support the potential for the clinical translation of this work, we investigated the potential use of BRCA gene expression following PI3K inhibition as a biomarker for treatment response to PARP inhibition.

## RESULTS

### The PI3K inhibitor BKM120 effectively blocked the proliferation of ovarian cancer cells with enhanced DNA damage response

**Figure 1:**
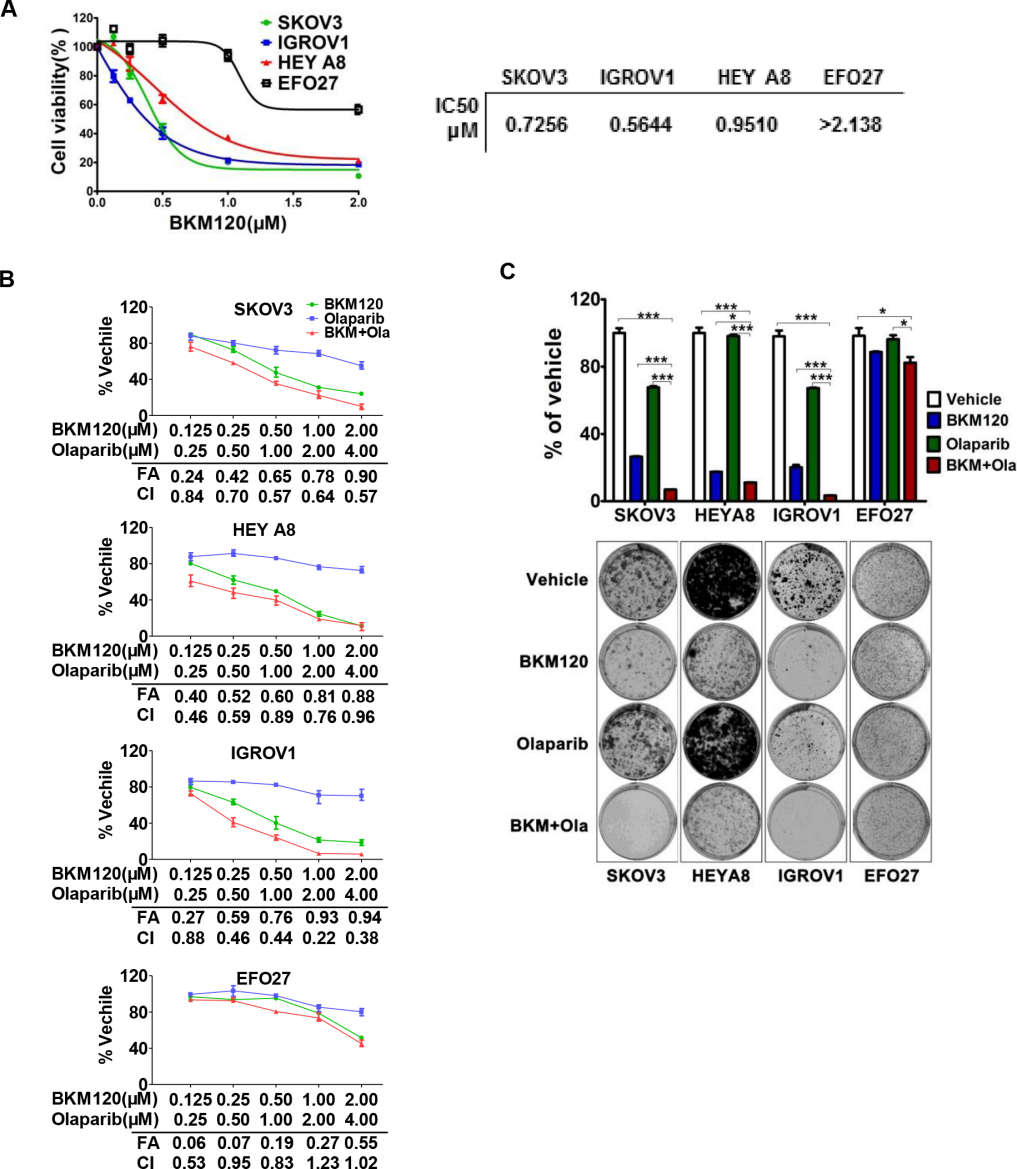
Responses of ovarian cancer cells to BKM120 and Olaparib as single-agents and in combination (**A**) IC50s of 4 ovarian cancer cell lines treated with BKM120 for 72 hours were determined using the CCK8 assay. (**B**) The four ovarian cancer cell lines were treated with BKM120 and Olaparib as single-agents or in combination for 72 hours and then subjected to CCK8 assay. The combined drug effect was analyzed using the CI equation and presented with FA combinations. (**C**) Ovarian cancer cells were treated with inhibitors as indicated for 10 days and then crystal violet stained. Mean ± S.D. for 3 independent experiments are shown. *P < 0.05; **P < 0.01; ***P < 0.001 (Student's *t* test).

BKM120 is a pan-class I PI3K inhibitor under active clinical development [21]. To determine the response of ovarian cancer cells to PI3K inhibition, we cultured a panel of 11 ovarian cancer cell lines in the presence of 1 μM BKM120 for up to 10 days. While BKM120 effectively inhibited the growth of majority of the ovarian cancer cell lines examined, we failed to identify an apparent association between a particular genetic alteration (*i.e. PIK3CA*, *K-Ras*, *p53*, *PTEN*, and *EGFR*) [22] and cellular response to BKM120 treatment (Figure S1A and S1B). To evaluate the efficacy of PI3K inhibitor BKM120 in ovarian cancer cells, we chose four *PIK3CA* mutant ovarian cancer cell lines (SKOV3, IGROV1, HEYA8, and EFO27) for further examination. Cell proliferation assay using Cell Counting Kit-8 (CCK-8) revealed that the IC50s of SKOV3, IGROV1 and HEYA8 for BKM120 were pronouncedly lower (0.7256 μM, 0.5644 μM, and 0.9510 μM, respectively) than that of EFO27 (more than 2.138 μM) (Figure 1A). We next assessed target inhibition by BKM120 treatment in these cancer cell lines. As expected, BKM120 markedly reduced the abundance of phosphorylated AKT protein (pAKT), a major effector of PI3K activation, in a time-dependent manner (Figure S2A). Accordingly, S6 ribosomal protein (S6RP) phosphorylation was also downregulated, indicating attenuated mTOR signaling (Figure S2A). Thus, consistent with its inhibitory effect on cell proliferation, the PI3K inhibitor BKM120 treatment resulted in attenuated PI3K/AKT/mTOR signaling in PIK3CA mutant ovarian cancer cells.

PI3K inhibition by BKM120 has been previously shown to incur DNA damage in breast cancer [17, 23], prostate cancer [19] and glioblastoma cells [24]. We next examined its effect on ovarian cancer cells. BKM120 treatment resulted in an increase in phosphorylation of histone 2AX on serine 139 (γH2AX), an indicator for double-stranded DNA breaks, in a time-dependent manner (Figure S2B). This increase also paralleled a decrease in the abundance of the homologous recombination repair protein RAD51 (Figure S2B) and phosphorylated AKT (Figure S2A). Similarly, PI3K inhibitor GDC-0941 as single-agent also led to an increase in γH2AX and a decrease in the abundance of RAD51 in ovarian cancer cell lines examined (Figure S3). Together, these results suggested an association between PI3K inhibition and enhanced DNA damage response in ovarian cancer cells.

### Combined use of BKM120 and Olaparib synergistically inhibited the growth of PIK3CA mutant ovarian cancer cells

Previous studies have indicated that cells deficient in homologous recombination (HR) repair are more susceptible to PARP inhibition [25, 26]. Given that BKM120 rendered PIK3CA mutated ovarian cancer cells more deficient in HR repair (Figure S2B), we hypothesized that combined inhibition of PI3K and PARP may lead to a stronger therapeutic effect than BKM120 as singe-agent. To test this hypothesis, we treated ovarian cancer cell lines with increasing concentrations of BKM120 and Olaparib, each alone and in combination for 72 hours. The effect of drug combination on proliferation inhibition was evaluated by CCK-8 assay followed by median-effect analysis [27]. Combined treatment with BKM120 and Olaparib resulted in a synergistic increase in proliferation inhibition at 0.5 fractions affected (FA) and synergistic combination index (CI) values of less than 1 over the majority of concentrations tested in SKOV3, IGROV1 and HEYA8 cells (Figure 1B). While concomitant treatment of EFO27 cells with BKM120 and Olaparib yielded CI values less than 1 over some but not all concentrations tested, the drug combination did not lead to any significant increase in the FA (Figure 1B). Thus, unlike the other three ovarian cancer cell lines examined, EFO27 cells were resistant to the growth inhibitory effects exerted by combined use of BKM120 and Olaparib.

We also determined the long-term effect of drug combination on ovarian cancer cells. PI3K inhibitor BKM120 as single-agent markedly reduced the proliferation of SKOV3, HEYA8 and IGROV1 cells (Figure 1C). In contrast, PARP inhibitor Olaparib exhibited only moderate inhibitory effect on the growth of SKOV3 and IGROV1 cells, but it had no impact on HEYA8 or EFO27 cells (Figure 1C). Furthermore, combined use of BKM120 and Olaparib nearly completely abrogated the growth of SKOV3 and IGROV1 cells, and to a lesser extent, in HEYA8 cells (Figure 1C). Similarly, PI3K inhibitor GDC-0941 as single-agent or in combination with Olaparib also yielded growth inhibitory effects on ovarian cancer cell lines examined, except for EFO27 cells (Figure S4). Together, these results indicated the therapeutic value of the combined PI3K and PARP inhibition on PIK3CA mutant ovarian cancer cells.

### BKM120 synergized with Olaparib to induce apoptosis in ovarian cancer cells harboring PIK3CA mutation

**Figure 2:**
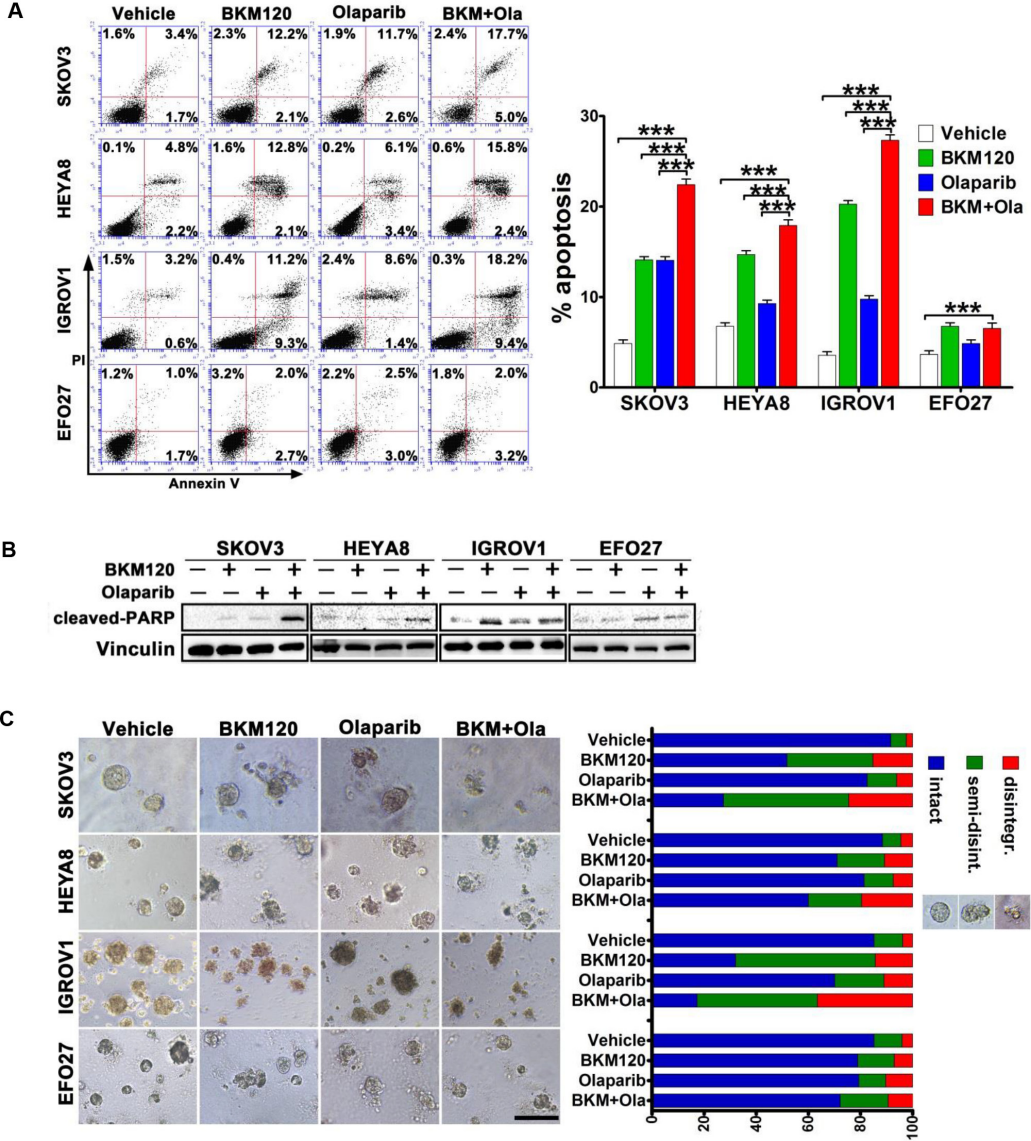
Effects of BKM120 and Olaparib as single-agents and in combination on the survival of ovarian cancer cells (**A**) Ovarian cancer cells were treated by BKM120 and Olaparib, each alone or in combination, for 48 hours. The percentage of apoptotic cells was determined by Annexin V and PI staining. Mean ± S.D. for three independent experiments are shown. ***P < 0.001 (Student's *t* test) (**B**) Western blot analysis of cleaved-PARP in ovarian cancer cells treated as indicated for 48 hours. Vinculin was used as a loading control. (**C**) Ovarian cancer cells as indicated were grown in 3D and treated with indicated drugs and quantitated for structural integrity after 72 hours drug treatment as described in Methods. Representative images of scored structures (intact, semi-disintegrated, disintegrated) are shown in right panel, scale bar 200 μm.

We next determined whether combined treatment with BKM120 and Olaparib would have an impact on apoptotic cell death [28]. While inhibition of PI3K or PARP alone yielded a moderate increase in apoptotic cell population (Annexin V-positive), combined treatment resulted in substantially increased apoptosis in SKOV3, IGROV1, and HEYA8 cells (Figure 2A). Consistent with this observation, combination treatment also effectively enhanced the abundance of cleaved PARP, a marker for active apoptosis, in SKOV3, IGROV1, and HEYA8 cells (Figure 2B). However, similar results were not found in EFO27 cells (Figure 2A and 2B).

Three-dimensional (3D) cell culture condition closely mimics an *in vivo* environment and can be used to examine how cancer cells respond to drug treatment [29]. The observation that combined use of BKM120 and Olaparib enhanced apoptosis in 2D culture condition prompted us to further determine the effect of drug combination on cell death in 3D assay condition. Inhibition of PI3K or PARP alone did not induce dramatic cell death, but combined treatment caused significant structural disintegration and cell death in SKOV3 and IGROV1 cells, and to a lesser extent in HEYA8 cells (Figure 2C). In contrast, the overall integrity of EFO27 cells was not significantly affected by combined treatment (Figure 2C). Together, these results suggested that combined use of BKM120 and Olaparib may have a therapeutic value in the treatment of ovarian cancer cells.

### BKM120 synergized with Olaparib to inhibit migration and invasion of PIK3CA mutant ovarian cancer cells

**Figure 3:**
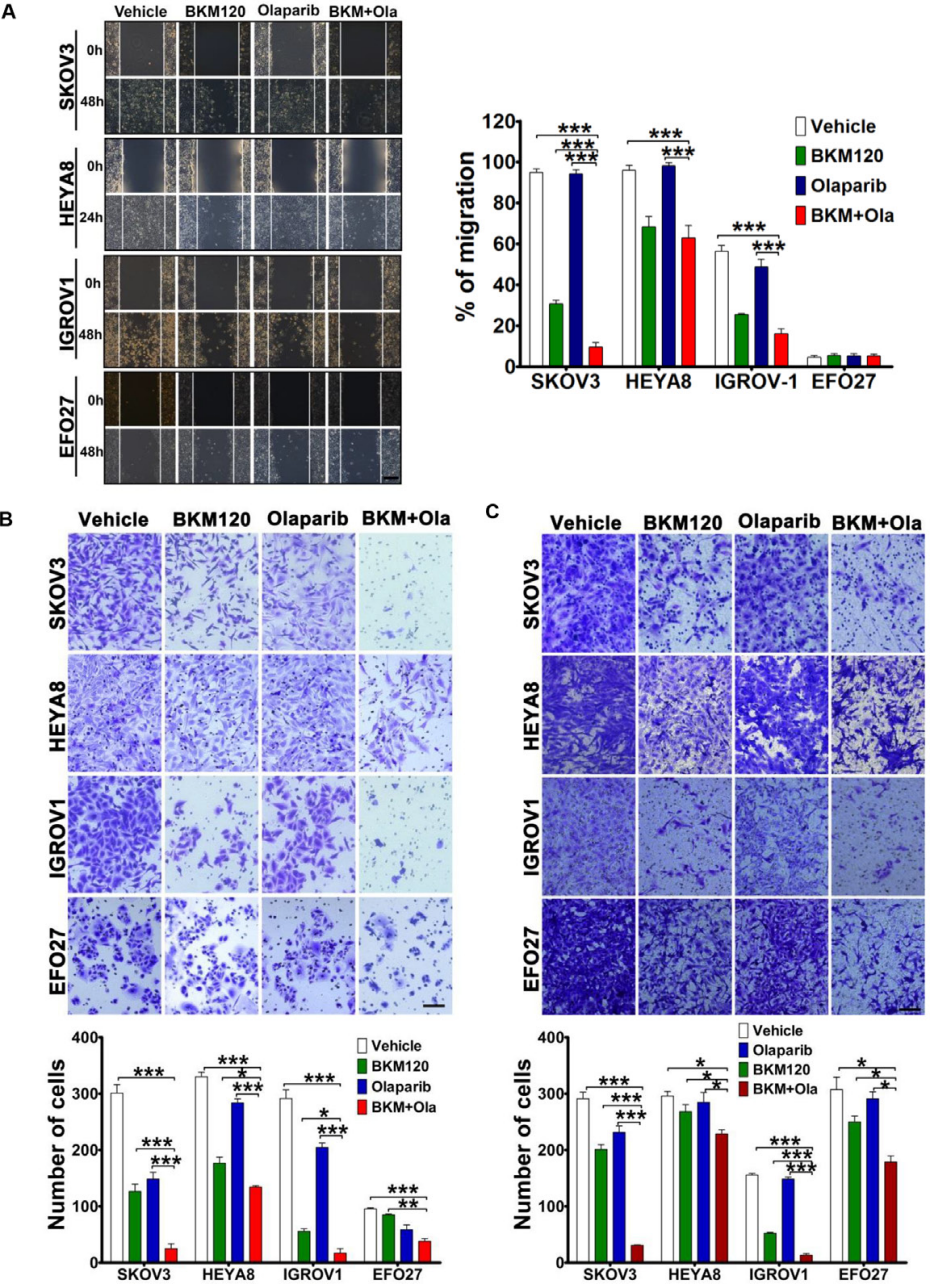
Effects of BKM120 and Olaparib as single-agents and in combination on migration and invasion of ovarian cancer cells (**A**) Cell migration potential was determined using a wound-healing assay. Images of the wound areas were shown at 0 and 24 h (HEYA8) or 48 h (SKOV3, IGROV1 and EFO27) (at magnification × 100, scale bar 200 μm). Mean ± S.D. for three independent experiments are shown. (**B**) Migration of ovarian cancer cells with the inhibitor treatment as indicated was assessed using Boyden chamber assay. Migrated cells on the underside of the filter were photographed and counted by phase contrast microscopy (at magnification × 200, scale bar 100 μm). Mean ± S.D. for three independent experiments are shown. (**C**) Ovarian cancer cells were subjected to a Matrigel invasion assay in the presence of inhibitor treatment as indicated. For each experiment, cell number was calculated as the total count from 10 random fields per filter (at magnification × 200, scale bar 100 μm). Mean ± S.D. for three independent experiments are shown. *P < 0.05; **P < 0.01; ***P < 0.001 (Student's *t* test).

Tumor cell migration and invasion are critical initiation steps in the process of ovarian cancer metastasis [30]. To examine the effect of combination treatment with BKM120 and Olaparib on cellular migration, we performed wound-healing assay with confluent monolayers of ovarian cancer cells. While BKM120 as single-agent led to partially filled gap, dual treatment with BKM120 and Olaparib resulted in significantly attenuated migration of SKOV3 cells (Figure 3A). BKM120 as single-agent or in combination with Olaparib displayed a similar inhibitory effect on migration of HEYA8 and IGROV1 cells (Figure 3A). Notably, Olaparib as single-agent had little effect on migration of all ovarian cancer cell lines tested. We also noted that EFO27 cells did not show migration in the assay conditions (Figure 3A).

We next used transwell assay to further examine the drug effect on migration. In line with the observation by wound-healing assay, combined use of BKM120 and Olaparib significantly inhibited the migration of SKOV3, HEYA8 and IGROV1 cells when compared to single agent-treated or vehicle-treated cells (Figure 3B). Dual treatment with BKM120 and Olaparib also inhibited the migration of EFO27 cells, but to a lesser degree (Figure 3B).

To determine the effect of drug combination on invasion of ovarian cancer cells, we used Matrigel invasion assay. While BKM120 as single-agent only moderately inhibited cell invasion, combined use of BKM120 and Olaparib significantly blocked the invasion of all four ovarian cancer cell lines examined (Figure 3C). Together, dual treatment of BKM120 and Olaparib resulted in strong inhibitory effect on migration and invasion potential of ovarian cancer cells.

### Combined use of BKM120 and Olaparib synergistically resulted in DNA damage of PIK3CA-mutant ovarian cancer cells

**Figure 4:**
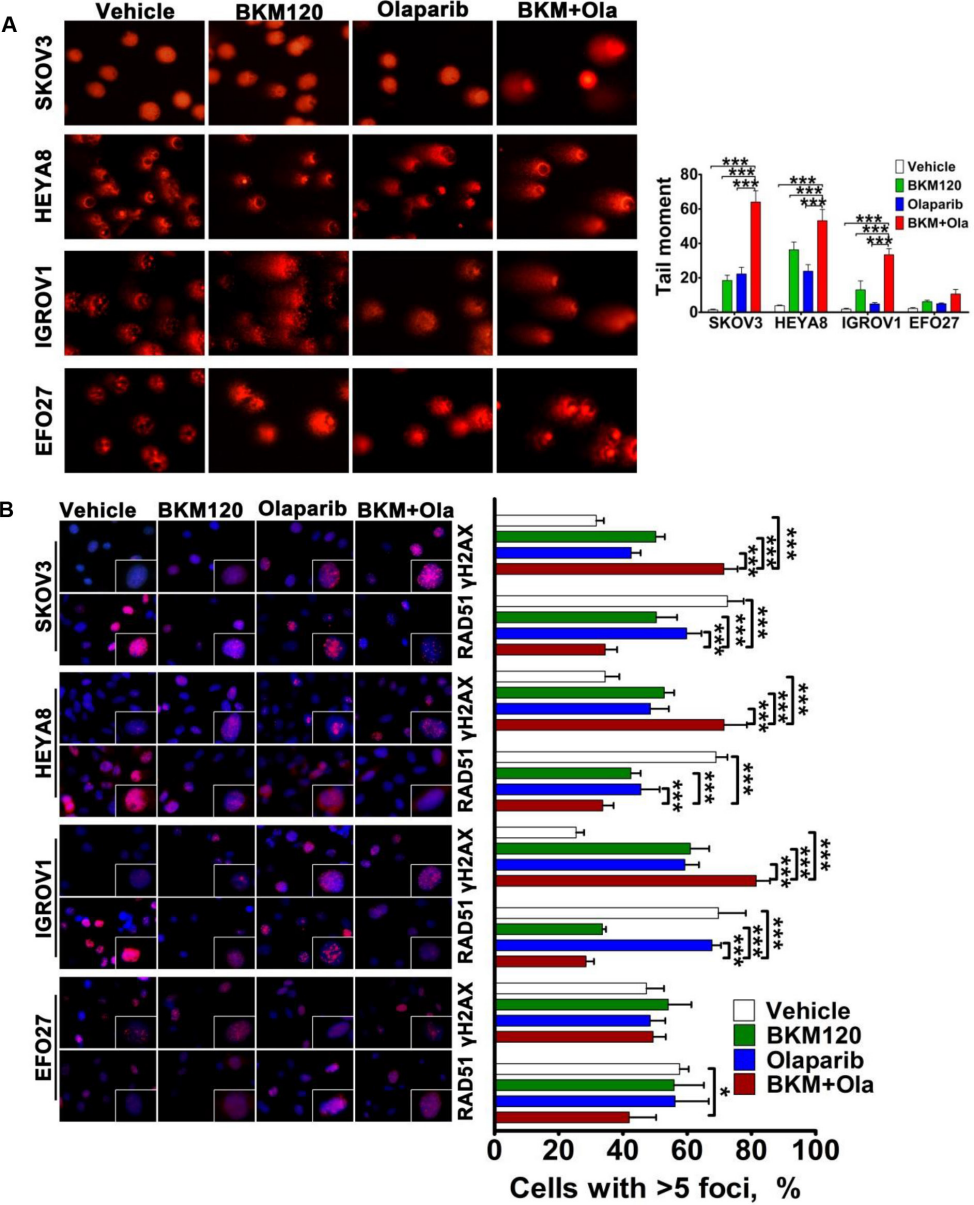
Effects of BKM120 and Olaparib as single-agents and in combination on DNA damage response in ovarian cancer cells (**A**) DNA damage in ovarian cancer cells was determined by comet assay. The ovarian cancer cells as indicated were treated with BKM120 and Olaparib as single-agents or in combination for 48 hours. Comet images × 200 taken by fluorescent microscope were shown. Tail moment was used to quantify DNA damage and evaluated by CASP software (CaspLab). Means ± S.D. of three independent experiments are shown. (**B**) Representative images of immunofluorescence staining of γH2AX and RAD51 in ovarian cancer cells treated as indicated for 48 hours (left panel). Cell nuclei were stained with DAPI. The percentage of cells with γH2AX and RAD51 foci was shown (right panel). Means ± S.D. of three independent experiments are shown. *P < 0.05; **P < 0.01; ***P < 0.001 (Student's *t* test).

As PI3K inhibitor BKM120 as single-agent led to an increase in γH2AX with a paralleled decrease in the abundance of homologous recombination protein RAD51 in ovarian cancer cells (Figure S2B), we next determined if additional use of PARP inhibitor Olaparib would further dampen cellular response to DNA damage. While BKM120 or Olaparib as single-agents moderately induced DNA damage as measured by tail moment, dual treatment with BKM120 and Olaparib resulted in substantially enhanced DNA damage in SKOV3, HEYA8 and IGROV1 cells (Figure 4A). Notably, EFO27 cells did not show DNA damage response in the assay conditions (Figure 4A).

To further assess the DNA damage response to dual inhibition of PI3K and PARP, we examined γH2AX foci and RAD51 recruitment by immunofluorescence staining. For SKOV3, HEYA8 and IGROV1 cells, combined use of BKM120 and Olaparib led to significantly increased number of γH2AX foci but remarkably reduced RAD51 recruitment when compared to single-agent treated or vehicle treated cells (Figure 4B and S5). While dual inhibition of PI3K and PARP resulted in exacerbated DNA damage response in these three cell lines, it has little effect on EFO27 cells (Figure 4B).

### Ovarian cancer cells that responded to the combination treatment displayed concomitantly decreased BRCA1/2 expression

**Figure 5:**
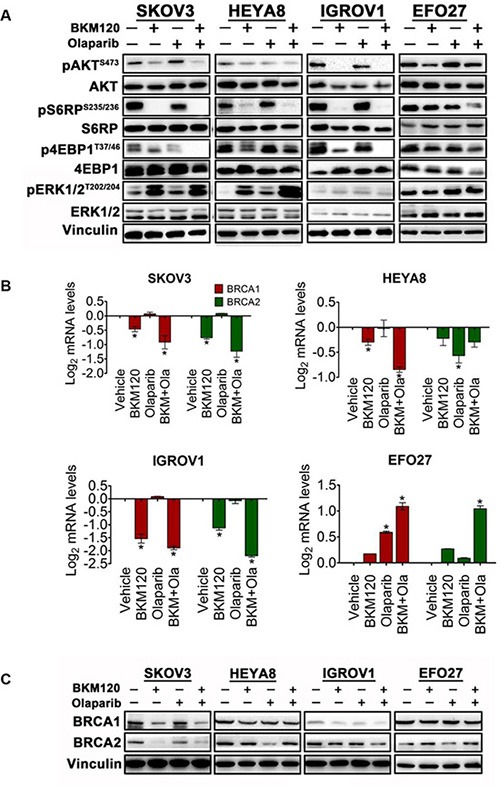
Effects of BKM120 and Olaparib as single-agents and in combination on PI3K/AKT/mTOR signaling and BRCA1/2 expression in ovarian cancer cells (**A**) Western blot analysis of proteins (pAKT, AKT, pS6RP, S6RP, p4EBP1, 4EBP1, pERK1/2 and ERK1/2) as indicated in ovarian cancer cells treated with BKM120 and Olaparib as single-agents or in combination for 48 hours. Vinculin was used as a loading control. (**B**) Quantitative reverse transcription PCR analysis of BRCA1 and BRCA2 expression in ovarian cancer cells treated with BKM120 and Olaparib as single-agents and in combination. Gene expression was normalized to GAPDH. Mean ± S.D. for three independent experiments are shown. *P < 0.05 (Student's *t* test). (**C**) Western blot analysis of BRCA1 and BRCA 2 proteins as indicated in ovarian cancer cells treated with BKM120 and Olaparib as single-agents or in combination for 48 hours. Vinculin was used as a loading control.

Our results revealed that combined use of Olaparib and BKM120 resulted in strong inhibitory effect on proliferation, migration and apoptosis with exacerbated DNA damage in PIK3CA mutant ovarian cell lines including SKOV3, IGROV1 and HEYA8 cells, but not EFO27 cells. We next set out to understand molecular mechanisms underlying the differential cellular responses to the combination treatment. We found that when compared to single-agent BKM120 treatment, combined use of BKM120 and Olaparib further reduced the abundance of phosphorylated AKT, S6RP and 4EBP1 proteins, downstream effectors of PI3K/AKT/mTOR signaling, in SKOV3, IGROV1 and HEYA8 cells (Figure 5A), suggesting that combined inhibition of PI3K and PARP could downregulate PI3K/AKT/mTOR signaling pathway. In contrast, only a rather weak effect on the PI3K/AKT signaling pathway was seen in EFO27 cells treated with BKM120 and Olaparib.

While all four ovarian cancer cell lines examined in this study carry PIK3CA mutations, they showed differential responses to combination treatment with BKM120 and Olaparib. Previous work by Ibrahim YH *et al.* reported that BRCA1/2 expression correlates with Olaparib sensitivity and that PI3K inhibition reduced BRCA1/2 expression via an ERK-dependent effect [23]. In line with this finding, we found that when treated with BKM120 alone or in combination with Olaparib, SKOV3 and HEYA8 cells exhibited marked downregulation of *BRCA1/2* expression with a concomitant increase in pERK (Figure 5A and 5B). Interestingly, while the level of ERK phosphorylation in IGROV1 cells was not affected by BKM120 or in combination with Olaparib (Figure 5A), the expression of *BRCA1/2* is dramatically downregulated (Figure 5B), suggesting *BRCA1/2* expression can also be modulated through an ERK-independent mechanism. Of note, the three responsive ovarian cancer cell lines (SKOV3, HEYA8 and IGROV1) exhibited markedly decreased *BRCA1/2* expression at both mRNA and protein levels upon BKM120 or BKM120/Olaparib treatment (Figure 5B and 5C). Conversely, the insensitive EFO27 cells showed increased *BRCA1/2* expression upon drug treatment (Figure 5B). Taken together, these results indicate that BRCA1/2 expression may be used to predict the efficacy of drug treatment in ovarian cancer cells.

### Combination treatment using BKM120 and Olaparib exhibited strong therapeutic effect on an intraperitoneal dissemination xenograft mouse model

**Figure 6:**
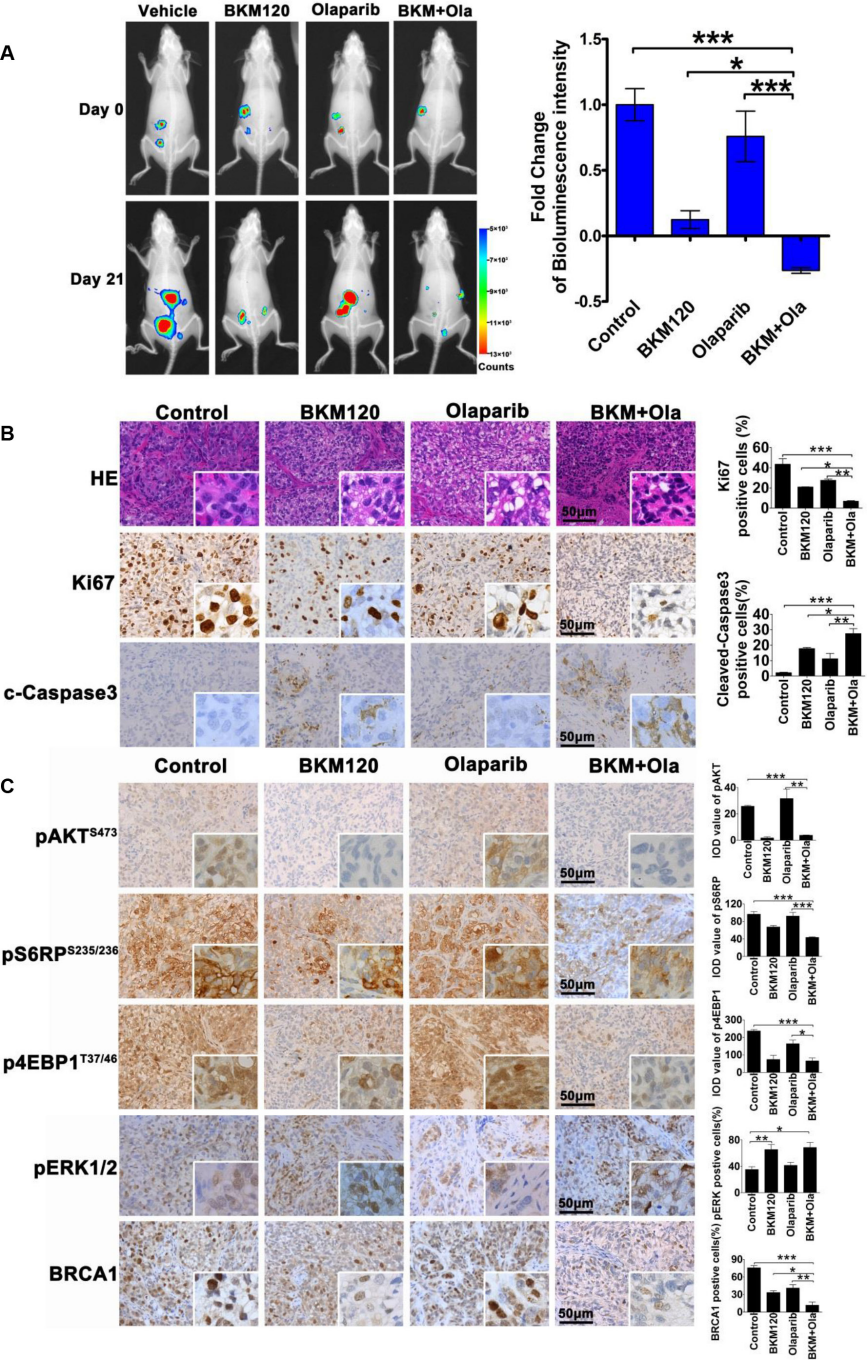
Bioluminescence imaging of SKOV3-Luc tumor response to BKM120 and Olaparib as single-agents and in combination SKOV3-Luc cells were injected into immunodeficient NOD/SCID mice via intraperitoneal injection. (**A**) Representative bioluminescence imaging of mice bearing intraperitoneally disseminated SKOV3-Luc tumors before (Day 0, baseline) and after inhibitor treatment as indicated for 21 days were shown. Fold change of bioluminescence intensity over baseline level was calculated and data were shown as mean ± S.E.M. (*n* = 6/treatment group). *P < 0.05; **P < 0.01; ***P < 0.001 (Student's *t* test). (**B**) Representative images for hematoxylin and eosin (H & E) staining and immunohistochemical staining analyses of Ki67 and cleaved-caspase 3 on SKOV3-Luc tumors isolated from mice treatment with either single-agents or the combination. (**C**) Representative images for immunohistochemical staining analyses of protein as indicated on the same tumor samples as in B. Scale bars, 50 μm.

To investigate the therapeutic effect of the combination treatment *in vivo*, we used an intraperitoneal dissemination xenograft mouse model of SKOV3 ovarian cancer cells. To facilitate the live imaging of tumor cell growth *in vivo*, we engineered SKOV3 cells to stably express luciferase (Luc), named SKOV3-Luc and then introduced these cells into immunodeficient NOD/SCID mice via intraperitoneal injection. Fourteen days post injection, bioluminescence imaging analysis was employed to ensure the establishment of disseminated tumors in the peritoneal cavity of mice injected with SKOV3-Luc cells (Figure 6A, top panel, baseline). These mice were then subjected to inhibitor treatment. When compared to the vehicle treatment, Olaparib has little effect on the luciferase signal (Figure 6A). In contrast, BKM120 as single-agent and to a more substantial degree when used in combined with Olaparib, led to attenuated luciferase signals. Apparently, the combination treatment caused a significant tumor cell killing effect in this mouse model. In line with these observations, tumors isolated from mice with combination treatment also exhibited large areas of necrotic or apoptotic cells (Figure 6B), indicating an effective therapeutic strategy. Consistently, we observed remarkably reduced proliferative index by Ki67 staining as well as significantly enhanced apoptotic cell death by cleaved caspase 3 staining (Figure 6B). Of note, we found that the combined use of BKM120 and Olaparib had little effect on mouse body weight (Figure S6) or the growth of normal mouse ovarian epithelial cells (Figure S7). Together, the combination treatment proposed in this study may be specifically effective on the treatment of ovarian cancer cells with little toxic effects on normal cells.

To assess inhibition of signaling pathways by combined use of BKM120 and Olaparib, we set out to analyze the xenograft tumors by immunohistochemical analysis. Remarkably, signals of pAKT, pS6RP and p4EBP1 were markedly reduced in tumors treated with dual BKM120 and Olaparib (Figure 6C). Together, these results suggested inhibition of PI3K/AKT/mTOR signaling at least in part account for the strong inhibitory effect on the growth of disseminated ovarian cancer cells by combined use of BKM120 and Olaparib. Of note, consistent with our finding by *in vitro* assays, tumors with the combination treatment revealed remarkably attenuated BRCA1 expression with concomitantly increased pERK signals when compared to vehicle or single-agent treated groups.

## DISCUSSION

The current study reported that combined inhibition of PI3K and PARP effectively blocked proliferation, survival, migration and invasion of a number of *PIK3CA* mutated ovarian cancer cells. Consistent with previous findings from preclinical studies of breast [17, 23] and prostate cancers [19], we also found that suppression of the PI3K signaling by BKM120 was accompanied by increased abundance of the double-stranded break marker γ-H2AX and decreased abundance of homologous recombination (HR) repair protein RAD51 in well-established cell models of *PIK3CA* mutant ovarian cancer. Our data revealed that PI3K suppression by BKM120 may render these ovarian cancer cells more deficient in HR repair and thus more dependent on the single-stranded DNA repair mechanism that relies on PARP.

It was shown previously that loss of PTEN, leads not only to activation of the PI3K pathway, but also to an accumulation of DNA DSBs and thus synthetic lethal interaction with PARP inhibition [25, 31–33]. Interestingly, the two PTEN-deficient ovarian cancer cell lines examined in the current study exhibited sensitivity toward PARP inhibitor Olaparib with differential responses. Indeed, in addition to mutational loss of PTEN, IGROV1 cells also bear mutations in *BRCA1*, *BRCA2* and *ARID1A* [22]. It is worth noting that deficiency of each of these gene products (*PTEN*, *BRCA1*, *BRCA2* and *ARID1A*) has been previously shown to render cancer cells responsive to PARP inhibition [23, 25, 31, 34], and may in this case collectively contribute to the superior sensitivity of IGROV1 cells to Olaparib when used in combination with PI3K inhibitor [34]. In contrast, while harboring mutational loss of *PTEN* [19, 25], EFO27 cells do not respond to PARP inhibition by Olaparib or PI3K inhibition by BKM120, either as single-agent or in combination, suggesting additional genetic alterations in this cell line may modulate their respective drug responses.

Phosphorylation of S6RP has been shown to confer resistance to PARP inhibitors in BRCA1-deficient breast cancers through regulation of DNA damage response and the HR repair process [35]. In the current study, we showed that PI3K inhibitor BKM120 as single-agent or in combination with Olaparib nearly completely abolished p-S6RP signals in the *in vitro* cell models of ovarian cancer (SKOV3, IGROV1, HEYA8) that respond well to drug treatment. In line with this, similarly strong inhibition of p-S6RP signals was observed in an intraperitoneal dissemination model of SKOV3 ovarian cancers following the combination treatment. However, it is worth noting while the combined use of Olaparib and BKM120 did lead to markedly reduced pS6RP signals, the remaining signals may still contribute to the resistance of EFO27 cells to the combination treatment.

It has been recently shown that concomitant BRCA1/2 downregulation following PI3K inhibition in breast cancer and prostate cancer is sufficient for the induction of HR deficiency, rendering cancer cells to acquire sensitivity to PARP inhibition [19, 23]. In the current study, we found that PI3K inhibition by BKM120 acts synergistically with PARP inhibition in abolishing the growth and survival of *PIK3CA* mutated ovarian cancer cells (SKOV3, HEYA8, and IGROV1) in *in vitro* assays as well as in an *in vivo* model of SKOV3 cells. Interestingly, the three examined ovarian cancer cell models that responded well to combination therapy also exhibited concomitantly diminished BRCA1/2 expression, which is consistent with the previously established synthetic lethal interaction between BRCA deficiency and PARP inhibition [23, 36]. Indeed, the degree of HR impairment induced by PI3K blockade may vary among different ovarian cancer cells; meanwhile, the sensitivity to dual PARP and PI3K inhibition may be dependent on the degree of concomitant BRCA1/2 downregulation that allows further impairment of DNA damage response/the HR repair process. Collectively, our data suggested that the combination of PI3K and PARP inhibitors represents a promising therapeutic approach for the treatment of *PIK3CA* mutated ovarian cancers. Concomitantly decreased BRCA1/2 expression might predict effective response of ovarian cancer cells to the combination treatment.

## MATERIALS AND METHODS

### Cell culture

SKOV3, PA1, SW626, CAOV3, OVCAR8, and OVCAR5 ovarian cancer cell lines were purchased from the American Type Culture Collection. The other lines reported here were available in our laboratories. SKOV3, PA1, SW626, CAOV3, OVCAR8, OVCA433, HEYA8 and OVCAR5 were cultured in DMEM supplemented with 10% fetal bovine serum (FBS), EFO27, IGROV1 and A2780 cells in RPMI-1640 supplemented with 10% FBS. Cells were incubated at 37°C in a 5% CO_2_-containing atmosphere.

### Cell proliferation assay

The effects of the given drugs on cell proliferation were measured as previously described [26]. Briefly, cells were trypsinized and seeded into six-well plates at 1000 cells/well density and then treated with indicated concentration of BKM120, combination (BKM120 plus Olaparib at the respective concentrations) or DMSO control. Cell culture medium containing drug or vehicle control was renewed every 3 days. The cells were incubated for about 10 days until colonies were large enough to be clearly discerned. Cells were fixed with cold methanol, stained with crystal violet (Sigma–Aldrich) and subsequently extracted with 10% glacial acetic acid. The optical density (OD) was measured at 570 nm by EnSpire^®^ Multimode Plate Readers (PerkinElmer).

### Growth inhibition assay and drug combination analysis

The cell counting kit-8 assay (Dojindo Molecular Technologies) was carried out according to the manufacturer's guidelines and as previously described [37]. IC50s were calculated from sigmoidal dose-response curves utilizing Prism. The combination effect was determined by the combination index (CI) method [27] using the Calcusyn software program (Biosoft). Data from cell viability assays were expressed as the fraction of growth inhibition by the individual drugs or the combination in drug-treated cells. Synergism was indicated by a CI values less than 1 and antagonism by a CI value more than 1 at 50% effect (Fraction Affected = 0.5).

### Immunoblotting and antibodies

Cells were lysed in RIPA buffer supplemented with protease and phosphatase inhibitors as described [35]. Cell lysates were cleared by centrifugation and proteins were resolved by electrophoresis and transferred to polyvinylidene fluoride (PVDF) membranes. Blots were probed with the following antibodies: pAKT(Ser473), pERK1/2, cleaved-PARP, pS6RP(Ser235/236), p4EBP1(Thr37/46) (Cell Signaling Technology), AKT, S6RP, ERK1/2, 4EBP1 (Proteintech) and vinculin (Sigma). Second antibody: Goat anti-Mouse IgG (HRP-conjugated, Thermo), Anti-rabbit IgG (HRP-conjugated, Cell Signaling Technology).

### Apoptosis measurement

Apoptosis in ovarian cancer cells were analyzed with Annexin V-FITC Apoptosis Detection Kit (Dojindo Molecular Technologies) according to manufacturer's instructions. Briefly, cultured cells were trypsinized with 0.25% trypsin without EDTA, and stained with Annexin V FITC and PI solution. Stained cells were then subjected to flow cytometry analysis on a BD Accuri™ C6 (BD Biosciences, NJ).

### 3D sphere culture

3D cell culture experiments were performed as previously described [38]. Briefly, ovarian cancer cells were seeded on 96-well plates coated with Matrigel (BD Biosciences). Cells were grown in DMEM or RPMI-1640 medium supplemented with 2% FBS and 2% Matrigel and allowed to grow for 4 days. Cells were then fed with media supplemented with 2% FBS and 2% Matrigel and drugs were also added at this time point. 3D structures were scored according to 3D structure integrity based on the resemblance to images shown in Figure 2C. Over 100 colonies were scored for each condition.

### Wound-healing, migration and invasion assay

Wound-healing assay was used to evaluate cell migration as described previously [26]. Briefly, cells were seeded in 24-well plates and grown until confluent state and then cells were scratched using sterile tips. Then the cell monolayer was rinsed twice with PBS to remove debris. Fresh culture medium was added with indicated drugs. The mean width of each scratch was measured using Image Pro Plus (Media Cybernetics).

For the transwell migration assay, cells treated with indicated drugs were seeded into the upper chambers in FBS-free medium at a density of 5 × 10^4^ cells per chamber and 600 μl of 10% FBS containing medium was placed in the lower chamber as a chemoattractant.

For the transwell invasion assay, 1 × 10^5^ cells per chamber were plated. Cells were allowed to invade through the Matrigel-coated inserts. After treatment, the cells were stained with 0.1% crystal violet solution. Cells that remained in the gel or attached to the upper side of the filter were removed with cotton swabs. Cells on the underside of the filter were examined by light microscope and counted using microscope at high-power.

### Comet assay

Cells were treated and harvested 48 h after drugs treatment, and 1.5 × 10^4^ cells from each sample were subjected to the neutral comet assay as described [39]. Following electrophoresis, the cells were stained with ethidium bromide and viewed using a fluorescence microscope (Leica DMI4000B). We analyzed 200 individual cell images from each group using Comet Assay Software Pect (CaspLab) software. Tail moment was defined and served as a quantitative measure of DNA damage.

### Immunofluorescence staining analysis of γH2AX and RAD51

Ovarian cancer cells were cultured on coverslips in 24-well plates for 48 hours in respective medium containing inhibitor. Cells were fixed with 4% paraformaldehyde, and blocked with a 5% BSA-phosphate buffer solution. The cells were then incubated with rabbit anti-RAD51 polyclonal antibody (Santa Cruz Biotechnology) or rabbit anti-γH2AX^Ser139^ polyclonal antibody (Cell Signaling Technology) overnight at 4°C. After washing with PBS, the cells were incubated with secondary antibodies and DAPI at room temperature. Images were acquired and quantified using an immunofluorescence microscope (Leica). The dynamics of γH2AX and RAD51 foci accumulation, as well as percentage of positive cells (more than 5 foci in one cell) were calculated based on analysis of about 200 cells.

### Quantitative reverse transcription polymerase chain reaction (qRT-PCR)

Total RNAs were extracted from cultured cells with TRIzol reagent (Life Technologies), according to the manufacturers’ instructions. Reverse transcription reactions were performed using PrimeScript^™^ RT reagent Kit with gDNA Eraser (Takara). To quantify the amount of transcripts, SYBR Green based qPCR was performed with PrimeScript^™^ RT Master Mix (Takara) using Real Time PCR System (Stratagene Mx3000p). The primers for human BRCA1 were 5′-GTCCCATCTGTCTGGAGTTGA-3′ (forward) and 5′-AAAGGACACTGTGAAGGCCC-3′ (reverse); BRCA2 5′-AAAGGACACTGTGAAGGCCC-3′ (forward) and 5′-TTCTTCCTCTCTTTCATTGCG-3′ (reverse). The specificity of amplicons was verified with melting curve analysis and the messenger levels were normalized using GAPDH, as an internal control.

### Intraperitoneal dissemination xenograft mouse model and *in vivo* bioluminescence imaging

SKOV3 cells expressing luciferase (SKOV3-Luc) were generated by retroviral transduction. 5 × 10^6^ SKOV3-Luc cells in 0.2 ml PBS were injected into the peritoneal cavity of six-week-old female NOD/SCID mice (Charles River Laboratory, China). Fourteen days post injection, bioluminescence imaging analysis was performed on mice using KODAK *In-Vivo* Imaging System. The tumor-bearing mice were randomized in groups of 6 mice per group. BKM120 was dissolved in 0.5% methylcellulose solution and administered via oral gavage at 30 mg/kg/day. Olaparib was dissolved in 10% hydroxypropyl-β-Cyclodextrin for intraperitoneal administration and dosed at 50 mg/kg/day. After treatment for 21 days, animals were bioluminescence imaged again. Data was analyzed using *In-vivo* image software. All animal experiments were carried out in accordance with the approval of the Animal Research Committee of Dalian Medical University.

### Histology and immunohistochemistry

Tumors were fixed in formalin overnight before paraffin embedding. Paraffin blocks were sectioned and stained with hematoxylin and eosin (H & E). Immunohistochemistry (IHC) was carried out using the antibodies Ki67 (Vector), pAkt (Ser473) (Invitrogen), pS6RP, p4EBP1, γH2AX, and pERK (Cell Signaling Technology), BRCA1 (Proteintech). All IHCs were done as described previously [40].

### Isolation and culture of ovarian epithelial cells

The mouse ovarian surface epithelial cells were obtained from mature mice by mild trypsinization as previously described [41]. Briefly, ovaries were removed from four adult female FVB mice and rinsed with Hank's balanced salt solution (HBSS). Ovaries were placed in a 15 ml conical culture tube containing 10 ml HBSS with 0.25% trypsin at 37°C for 30 minutes. The epithelial cells were pelleted by centrifugation, resuspended in 2 ml complete medium (DMEM supplemented with 4% FBS, 1× penicillin-streptomycin, 1× insulin-transferrin-selenium).

### Statistical analysis

Quantitative results were analyzed by two-tailed unpaired Student's *t* test. *P* < 0.05 was considered statistically significant. All statistical analyses were performed using the GraphPad Prism 5.0 (San Diego, CA, USA).

## SUPPLEMENTARY MATERIALS FIGURES


