# Supplementary material for: Effective use of PI3K inhibitor BKM120 and PARP inhibitor Olaparib to treat PIK3CA mutant ovarian cancer
Source: Oncotarget. 2016 Feb 21;7(11):13153–66. doi: 10.18632/oncotarget.7549 (PMC4914348; doi:10.18632/oncotarget.7549)
Supplement: Supplementary file 1 [file oncotarget-07-13153-s001.pdf]

Effective use of PI3K inhibitor BKM120 and PARP inhibitor Olaparib to treat PIK3CA mutant ovarian cancer

Supplementary Materials

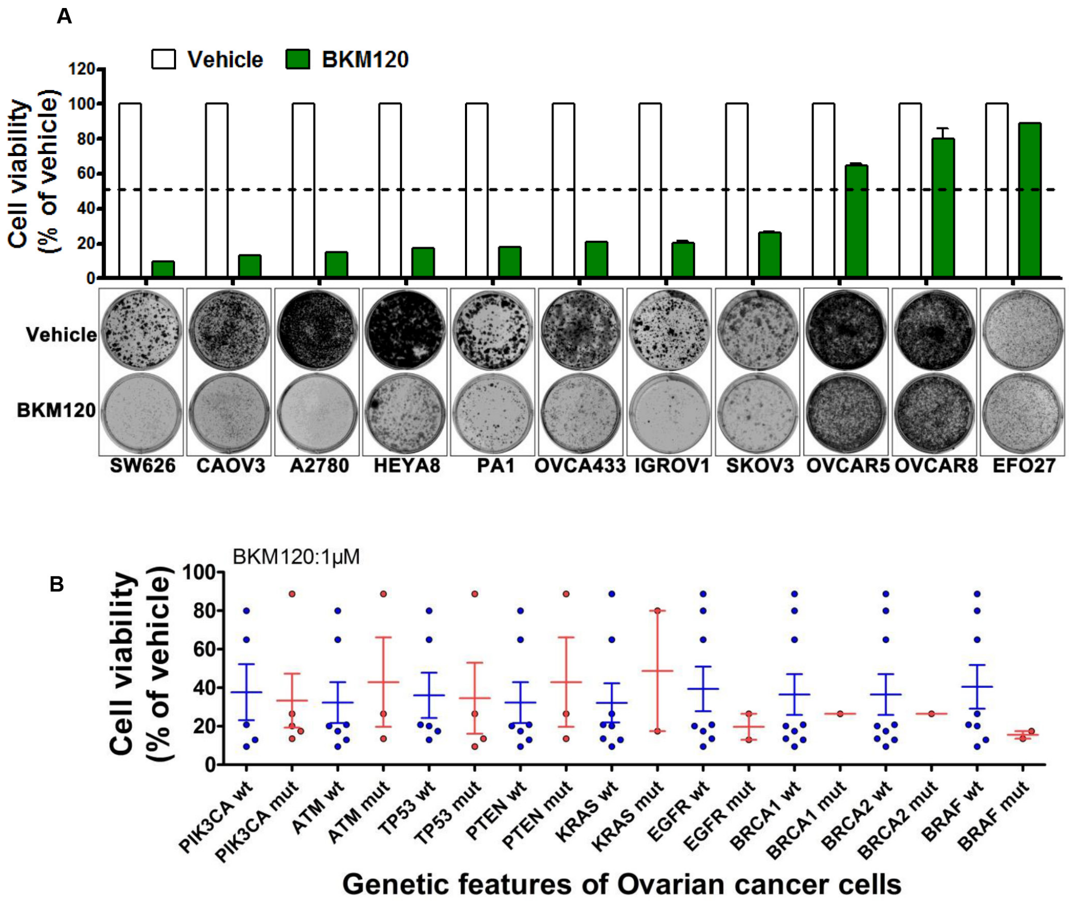

Supplementary Figure S1: Responses of ovarian cancer cells to BKM120 as a single agent. (**A**) Ovarian cancer cell lines as indicated were cultured in plates with or without 1 μM BKM120. On day 10, the plates were stained with crystal violet and representative images were shown. Mean ± S.D. for three independent experiments were shown. (**B**) The data of genetic features of ovarian cancer cells derived from COSMIC (http://www.sanger.ac.uk/perl/genetics/CGP/cosmic).

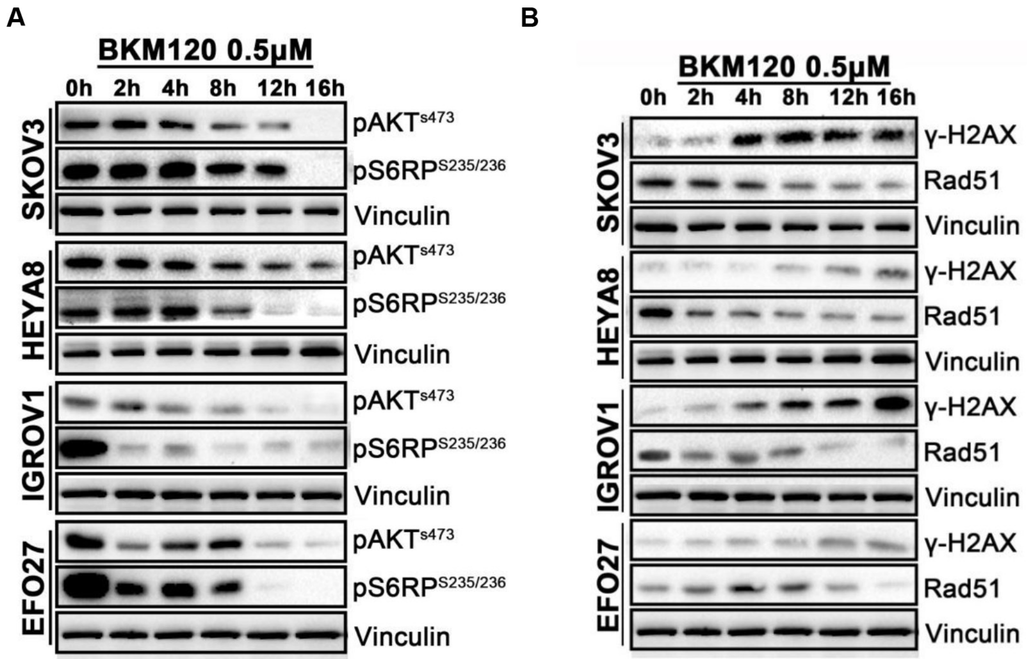

Supplementary Figure S2: Effects of BKM120 on PI3K/AKT/mTOR signaling and DNA damage response in ovarian cancer cells. (**A** and **B**) The given cell lines were treated with 0.5 μM BKM120 for different times as indicated. Western blot analysis of proteins (pAKT, pS6RP, γH2AX, and RAD51) in ovarian cancer cells treated with BKM120. Vinculin was used as a loading control. Representative images from three independent experiments were shown.

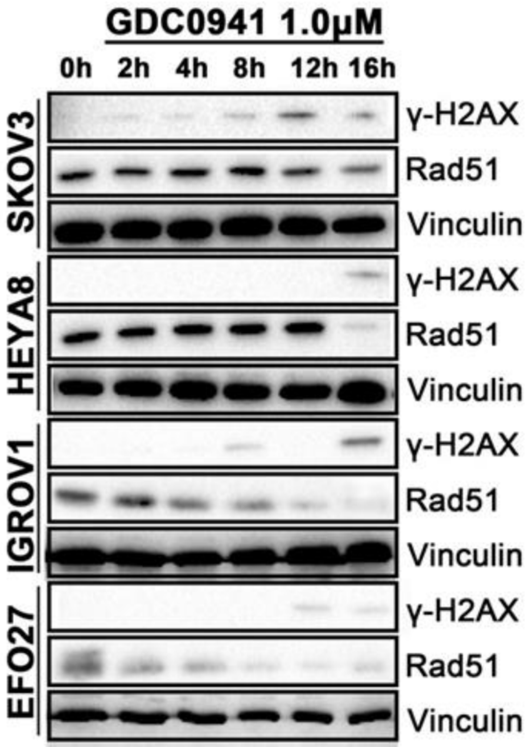

Supplementary Figure S3: Effects of GDC0941on DNA damage response in ovarian cancer cells. The given cell lines were treated with 1.0 μM GDC0941 for different times as indicated. Western blot analysis of proteins (γH2AX, and RAD51) in ovarian cancer cells treated with GDC0941. Vinculin was used as a loading control. Representative images from three independent experiments were shown.

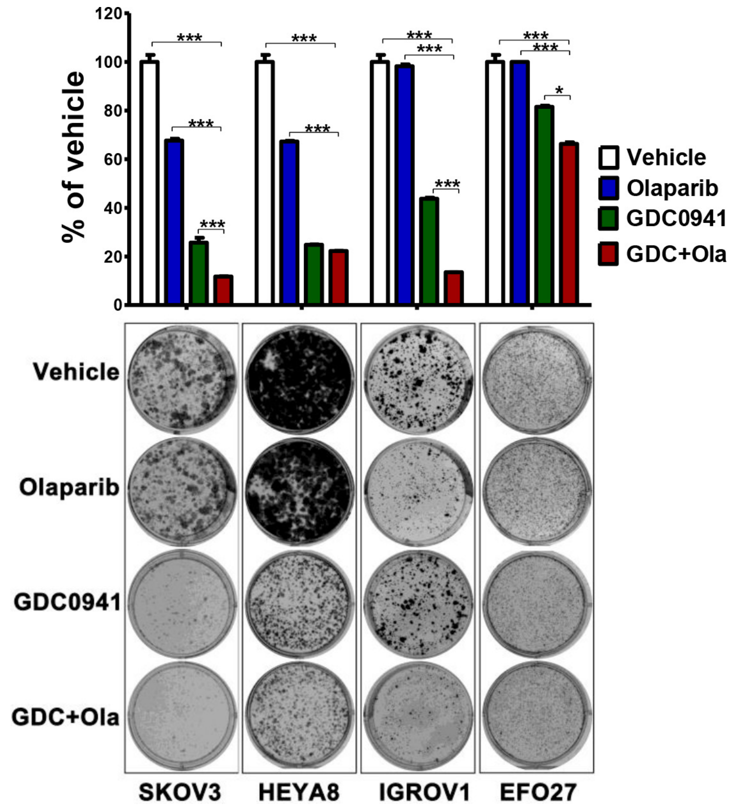

Supplementary Figure S4: Responses of ovarian cancer cells to GDC0941 and Olaparib as single agents and in combination. Ovarian cancer cell lines as indicated were cultured in plates with or without 1 μM GDC-0941, 2 μM Olaparib or in combination. On day 10, the plates were stained with crystal violet and representative images were shown. Mean ± S.D. for three independent experiments were shown. *$P < 0.05$; **$P < 0.01$; ***$P < 0.001$.

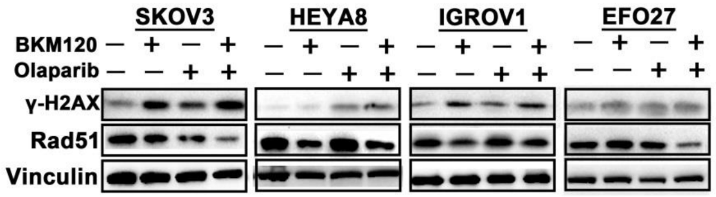

Supplementary Figure S5: Effects of BKM120 and Olaparib as single agents and in combination on DNA damage response in ovarian cancer cells. Western blot analysis of proteins (γH2AX and RAD51) as indicated in ovarian cancer cells treated with BKM120 and Olaparib as single agents or in combination for 48 hours. Vinculin was used as a loading control.

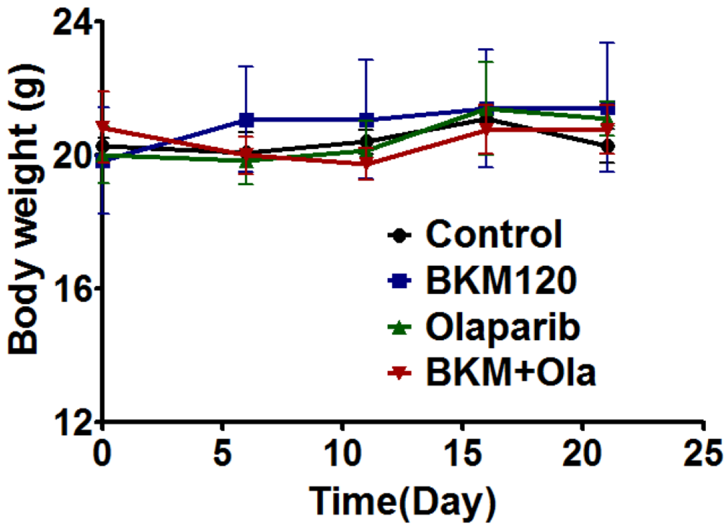

Supplementary Figure S6: Body weight measurement of mice bearing xenografts of SKOV3-Luc as shown in Figure 6. Data are means ± S.E.M. (n = 6 per treatment group). Treatment began on day 0. There was no statistical difference in the body weights of either group of mice throughout the 3-week study.

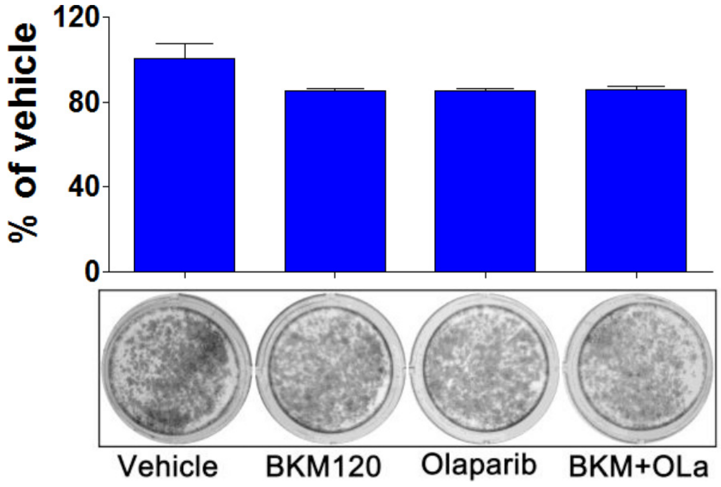

Supplementary Figure S7: Responses of mouse ovarian surface epithelial cells to BKM120 and Olaparib as single agents and in combination. Ovarian surface epithelial cells isolated from mouse ovaries were cultured in plates with or without 1 μM BKM120, 2 μM Olaparib or in combination. On day 10, the plates were stained with crystal violet and representative images were shown. Mean ± S.D. for three independent experiments were shown.
